# A Review of Bioinsecticidal Activity of *Solanaceae* Alkaloids

**DOI:** 10.3390/toxins8030060

**Published:** 2016-03-01

**Authors:** Szymon Chowański, Zbigniew Adamski, Paweł Marciniak, Grzegorz Rosiński, Ender Büyükgüzel, Kemal Büyükgüzel, Patrizia Falabella, Laura Scrano, Emanuela Ventrella, Filomena Lelario, Sabino A. Bufo

**Affiliations:** 1 Department of Animal Physiology and Development, Faculty of Biology, Adam Mickiewicz University, Umultowska 89 Str., Poznań, 61-614, Poland; pmarcin@amu.edu.pl (P.M.); rosin@amu.edu.pl (G.R.); 2 Electron and Confocal Microscope Laboratory, Faculty of Biology, Adam Mickiewicz University, Umultowska 89 Str., Poznań, 61-614, Poland; 3 Department of Molecular Biology and Genetics, Faculty of Arts and Science, Bulent Ecevit University, Zonguldak, 67100, Turkey; endericen@hotmail.com; 4 Department of Biology, Faculty of Arts and Science, Bulent Ecevit University, Zonguldak, 67100, Turkey; buyukguzelk@hotmail.com; 5 Department of Science, University of Basilicata, Via dellAteneo Lucano 10, Potenza, 85100, Italy; patrizia.falabella@unibas.it (P.F.); emanuelaventrella@libero.it (E.V.); filomenalelario@hotmail.com (F.L.); sabino.bufo@unibas.it (S.A.B.); 6 Department of European Culture, University of Basilicata, Via S. Rocco 1, Matera, 75100, Italy; laura.scrano@unibas.it

**Keywords:** insect physiology, pest control, bioinsecticides, *Solanaceae* secondary plant metabolites

## Abstract

Only a small percentage of insect species are pests. However, pest species cause significant losses in agricultural and forest crops, and many are vectors of diseases. Currently, many scientists are focused on developing new tools to control insect populations, including secondary plant metabolites, e.g., alkaloids, glycoalkaloids, terpenoids, organic acids and alcohols, which show promise for use in plant protection. These compounds can affect insects at all levels of biological organization, but their action generally disturbs cellular and physiological processes, e.g., by altering redox balance, hormonal regulation, neuronal signalization or reproduction in exposed individuals. Secondary plant metabolites cause toxic effects that can be observed at both lethal and sublethal levels, but the most important effect is repellence. Plants from the *Solanaceae* family, which contains numerous economically and ecologically important species, produce various substances that affect insects belonging to most orders, particularly herbivorous insects and other pests. Many compounds possess insecticidal properties, but they are also classified as molluscides, acaricides, nematocides, fungicides and bactericides. In this paper, we present data on the sublethal and lethal toxicity caused by pure metabolites and crude extracts obtained from *Solanaceae* plants. Pure substances as well as water and/or alcohol extracts cause lethal and sublethal effects in insects, which is important from the economical point of view. We discuss the results of our study and their relevance to plant protection and management.

## 1. Introduction

Humanity faces many problems that arise from its rapidly increasing population and one is the provision of the population with good quality food that is accessible for all [1]. There are various strategies that may be used to increase crop yield and improve food production, but various problems remain. Among them, the destruction of crops by pests is one of the most difficult, especially in developing countries, and although pests make up only a small percentage of insects, they cause significant losses to agricultural and forest crops, such as contributing to 20% annual loss of cereal crops [2]. The most voracious of the insect pests are *Lepidoptera* larvae, which have huge nutritional needs and are thus the most detrimental to food production [3]. For example, the diamondback moth, *Plutella xylostella* (L.), which is one of the major pests of *Brassicaceae* crop plants, causes annual losses of between USD 1.3 billion and USD 2.3 billion [4]. Moreover, many insect species are vectors of diseases that lead to millions of human deaths each year [3]. For example, malaria, which is transmitted by mosquitoes, kills over 600 thousand people annually around the world [5]. Therefore, the search for effective tools to control insect populations is one of the most intensively developing fields of research. Presently, the most common way to control insect pests is through the use of synthetic pesticides, but they negatively impact the natural environment [6]. These compounds have a wide spectrum of activities against diverse groups of insects and can almost completely remove pests from agroecosystems. However, although the immediate impacts and high efficiency of synthetic insecticides seem positive, there is no way to limit their action to only agricultural areas. The negative effects of synthetic insecticides are due to their insufficient selectivity, accumulation in the environment and food chains, long persistence, disturbance of the balance of ecosystems [7,8], and high socio-economic costs (poisoning as well as water and food contamination) [9,10] as well as the development of resistance in pest species [11]. Additionally, more selective pesticides are more expensive, so the inexpensive, nonselective pesticides are primarily used in developing countries [12]. These problems have forced humankind to search for alternatives to these compounds, and the demands of agriculture include inexpensive insecticides that cause the least amount of damage to the environment.

Integrated pest management (IPM) involves various plant protection strategies with an emphasis on a variety of biological control agents such as predatory animals, plant-derived substances, crop rotation and mechanical damage to pests. The use of highly toxic insecticides must be limited, so substances with lower toxicity should be used first. Such a strategy may significantly decrease the amount of pesticides released to the environment, although chemical approaches are and will continue to be a very important component of crop protection.

Are there any alternatives to synthetic insecticides? Among other candidates, secondary plant metabolites, such as alkaloids, glycoalkaloids, terpenoids, organic acids or alcohols, are regarded as promising sources of plant-protecting substances [13,14]. These compounds are produced by a variety of plant species in practically all their organs, and they are one of the most important lines of plant defense against pests. The range of cellular targets for these substances is very wide and covers metabolic pathways, macromolecules (e.g., proteins or nucleic acids) and organelles (e.g., biological membranes or nuclei) and they affect the functioning of entire organisms, such as when they bind to receptors, and impair nervous system function (for review, see Wink [15]). More importantly, especially in developing countries, effective biological controls are cost-effective. For example, on average, pesticides provide about $4 in benefits for every dollar invested in pesticide control. Furthermore, pest control using biological agents is more profitable, ranging from $30 to $100 for each dollar invested [16].

The increase in the content of secondary metabolites in plant organs is a common effect of pest invasion, and it can be said that plants use these compounds as chemical weapons [17]. The strategies vary; these compounds can lower the attractiveness of a plant to insects, make a plant unpalatable [18], or attract insects to poison them, *i.e.*, a lure-and-kill strategy [19], *etc.* However, the primary result is a reduction in the attractiveness of plants to insects. Additionally, the short half-life of natural insecticides in the environment is one of their most important attributes [11,20], and due to pleiotropy and their broad physiological activity, pests do not easily develop resistance to them. One of the reasons is that biopesticides usually have multiple modes of action due to their complex chemical composition, so insects are affected by many different compounds, which reduces the probability of developing resistance to all the chemicals [19]. The evolutionary co-existence of plants and insects has led to the development of complicated interactions among these two groups of organisms including the above-mentioned repellence and acute toxicity of natural insecticides that also affect plant pollination and insect reproduction and can be acquired and used by herbivorous insects against predators and parasitoids (for a review, see Ibanez, Gallet and Despres [14]).

The secondary metabolites produced by plants have a wide spectrum of activity; they affect insects at the cellular, tissue and organismal level. In general, their action disturbs the cellular and physiological processes responsible for maintaining homeostasis, and they can provoke sublethal changes within various tissues and organs, which can ultimately lead to death. However, secondary metabolites also have sublethal implications, such as reduced fecundity, reduced viability or deformities in parental and filial generations. In addition, these compounds reduce the number of individuals in populations both directly (as a result of death) but also, or even primarily, indirectly. Secondary plant metabolites can disturb development, lead to malformations or malfunctions, extend the duration of developmental stages [21,22,23,24,25] or act as repellents [26,27]. These phenomena may influence reproduction in the affected populations or cause the migration of herbivores away from plants rich in these compounds, which can decrease the number of pests in a given area.

One of the plant taxa that produce highly toxic compounds is the *Solanaceae* family. In this family, in which some of the most poisonous plants can be found [28,29,30], substances and extracts obtained from these plants are also widely used as pesticides [31]. Additionally, many economically important plants, e.g., potato, tomato and tobacco, belong to this family and are widely harvested. Therefore, unused plant organs, which contain biologically active substances, can be easily used to obtain relatively inexpensive sources of biological insecticides. In this review, we focus on the activity of pure alkaloids and extracts from *Solanaceae* against insects; these compounds have the potential to be applied in agricultural pest control programs.

## 2. *Solanaceae* Secondary Metabolites

The production of biologically active metabolites is widespread in the plant kingdom, and plant metabolic products have been used by humans for ages. The *Solanaceae* family belongs to the most important plant taxa, particularly in terms of food production (e.g., tomatoes and potatoes), and its members are used in medicine (e.g., deadly nightshade, jimson weed) or as drugs (e.g., tobacco) [32]. *Solanaceae* plants have an enormous potential to deliver new chemicals for crop protection; more and more of these compounds, or mixtures of these compounds, are being identified as pest control agents, especially against insects, fungi and mites [33].

Secondary metabolites can be classified according to their chemical structure, composition, solubility in different solvents, or on the basis of their synthesis pathway. A simple classification, based on chemical structure, includes three main groups: terpenes (composed almost entirely of carbon and hydrogen and including plant volatiles, cardiac glycosides, carotenoids and sterols), phenolics (with the common feature of having one or more phenol rings and including phenolic acids, coumarins, flavonoids, tannins and lignin), and nitrogen-containing compounds (extremely diverse, including alkaloids and glucosinolates).

Alkaloids are the most common biologically active compounds within the *Solanaceae* family, and they are strongly physiologically active in mammals, including humans. The effects of these plants can range from being stimulatory, *i.e.*, narcotic, to toxic and can cause death, even in very low doses [34,35,36]. Tomato and potato are the best known and most widely used plants in this group, and they constitutively synthesize low levels of many different glycoalkaloids. However, under stress (e.g., the emergence of herbivores), they increase the synthesis of these compounds, and in the case of potato (*Solanum tuberosum*), glycoalkaloids function as natural defense substances against pathogens and insects [37]. The common potato contains two major toxic steroidal glycoalkaloids, α-solanine and α-chaconine, which are biosynthetically derived from cholesterol, [38] and both are normally present in tubers in small amounts (<5 mg/100 g of tuber fresh weight). These natural toxicants (stress metabolites) have insecticidal and fungicidal properties and because naturally occurring pesticides are often synthesized when plants are under stress, injured plant tissues instigate the synthesis of higher concentrations of these compounds. Hlywka *et al.* [39] found that tubers from plants subjected to defoliation by the Colorado potato beetle (*Leptinotarsa decemlineata* Say) contained higher glycoalkaloid concentrations than tubers from control plants. In addition, potatoes that have been exposed to light in the field or during storage may become green due to an accumulation of chlorophyll, which may affect only the surface (peel) or may extend into the flesh. Exposure to light is only one of the stress factors affecting potato tubers; other pre- or post-harvest stress factors include mechanical damage, improper storage conditions either as a tuber or after partial food processing, and sprouting [38]. As a result of any of these stressors, there can be a rapid increase in the concentration of α-solanine and α-chaconine which gives the potatoes a bitter taste. Glycoalkaloids are formed in the parenchyma cells of the periderm and the cortex of tubers, as well as in areas of high metabolic activity such as the eye regions. These compounds are unevenly distributed throughout the potato, with a large concentration in the peel [40], and some cultivars are more prone to developing elevated levels of glycoalkaloids than others [41].

### 2.1. Solanaceae Alkaloids—Chemical Structure

Alkaloids constitute a very large group of pharmacologically active, nitrogen-containing compounds; in fact, more than 12,000 alkaloids have been described. They are defined as basic compounds synthesized by plants that contain one or more heterocyclic nitrogen atoms; they are derived from amino acids, and a few of them from terpene or purine and pyrimidine bases (pseudo-alkaloids). The class name means alkali-like, and *alkaloids* are, in fact, organic bases similar to the alkalies (inorganic bases). Examples of well-known alkaloids include strychnine, cocaine, caffeine, nicotine, α-solanine and α-tomatine [42].

The broad alkaloid group is divided into a number of subgroups that share similar structures, and closely related plant species generally contain alkaloids with related chemical structures. *Solanaceae* is a widespread family of species rich in alkaloids, including tropane alkaloids, glycoalkaloids, pyrrolizidine and indole alkaloids, which are naturally produced as a defense mechanism against insects, predators and disease [33].

Steroidal glycoalkaloids are a group of glycosidic derivatives of nitrogen-containing steroids that are produced in more than 350 plant species, mainly those in the *Solanaceae* and *Liliaceae* families [43]. They consist of a C27 cholestane skeleton (aglycone) to which a carbohydrate moiety of one to five monosaccharides is attached at the 3-OH position of the aglycone [43,44]. The carbohydrate moiety consists of different combinations of D-glucose, D-galactose, D-xylose and L-rhamnose, and to date, more than 75 naturally occurring aglycone structures are known [43]. Because nitrogen is inserted into a non–amino acid residue, these compounds belong to a subgroup of pseudo-alkaloids, *i.e.*, the isoprenoid alkaloids. The structural variation in plant glycoalkaloids is limited to two main groups that are based on the skeletal type of the aglycone [45]. The spirosolan type is constituted by a tetrahydrofuran and piperidine spiro-linked bicyclic system with an oxa-azaspirodecane structure (as in solasodine), and the solanidane type is formed by an indolizidine ring where tertiary nitrogen connects the two rings (as in solanidine). In these structures, nitrogen can be attached as a primary NH_2_ group (free or methylated), which forms simple steroidal bases, ring-closed to skeletal (as a secondary NH) or in two rings as a tertiary *N*, which often influences the chemical character of the compound [46]. All types of glycoalkaloids can contain double bonds and hydroxyls (OH) in various positions, and at least 90 structurally unique steroidal alkaloids have been identified in over 350 *Solanum* species [45].

**Figure 1:**
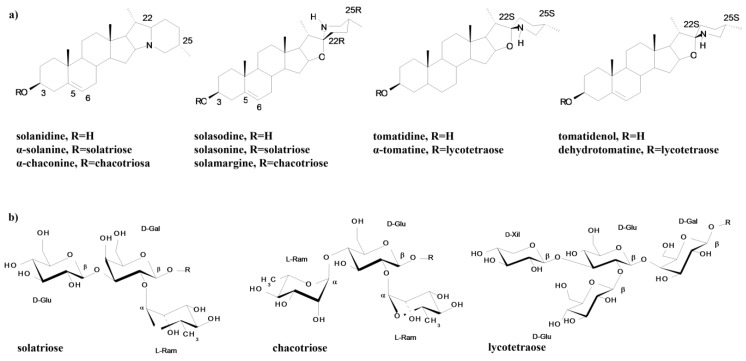
Structure of the major glycoalkaloids of the potato, eggplant and tomato: aglycones (**a**) and carbohydrate moieties (**b**).

The major components of the glycoalkaloid family are α-solanine and α-chaconine in potato plants (*S. tuberosum* L.), and solasonine and solamargine in eggplants (*Solanum melongena* L.), whereas α-tomatine and dehydrotomatine are spirosolane-type glycoalkaloids that occur in tomato plants (*Lycopersicon esculentum* Mill.) [24,47] (Figure 1). The potato plant produces the glycoalkaloids, α-chaconine and α-solanine, which share a common aglycone, solanidine, to which a trisaccharide moiety, either chacotriose (α-chaconine) or solatriose (α-solanine), is attached (Figure 1). Similar units are attached to the aglycone, e.g., solasodine in the eggplant, thereby producing the glycoalkaloids solamargine and solasonine (Figure 1). The tomato plant produces the compounds α-tomatine and dehydrotomatine, which differ in only the presence or absence of a double bond in the ring structure. In domestic potato plants, α-solanine and α-chaconine are usually the two dominant glycoalkaloids [47], but several other glycoalkaloids may also be found in wild species [48].

In the environment and in insects after penetration in the organism, the glycoalkaloids may undergo acidic or enzymatic hydrolysis whereby the side carbohydrates chain is partly or fully removed. Hydrolytic removal of all three sugar residues from α-chaconine results in the formation of the aglycone solanidine, which lacks a carbohydrate side chain [49].

Many species of *Solanaceae* also produce another important class of secondary metabolites called tropane alkaloids that have pharmacologically important properties but may also be poisonous. Tropane alkaloids consist of over 200 known compounds that share a tropane skeleton as a common structural feature and are characterized by a pyrrolidine and a piperidine ring sharing a single nitrogen atom and two carbons atoms. These alkaloids are commonly found in plants of different families: *Solanaceae*, *Erythroxylaceae*, *Convolvulaceae*, *Proteaceae*, *Euphorbiaceae*, *Rhizophoraceae* and *Brassicaceae* [50]. In recent decades, the well-known effects of tropane alkaloids as anticholinergic and anaesthetic agents have stimulated considerable interest in the biosynthetic pathway that leads to tropane alkaloids [51,52]. The diversity of tropane alkaloids is achieved by elaboration of the tropane skeleton, which originates from ornithine, acetate and methionine through different types of modifications. Tropane alkaloids are found in all plant parts, with the highest concentrations in roots and seeds in proportions that vary among species, time of year, location, and plant organs.

A few important members of the tropane alkaloids are atropine, hyoscyamine and scopolamine, and high concentrations of these alkaloids have particularly been found in *Datura stramonium* L. and *Datura ferox* L. as well as in *Datura innoxia* Mill. Atropine is a racemic mixture of the two enantiomeric forms of hyoscyamine, and hyoscyamine is active only in the L-enantiomeric form. Scopolamine, which acts as an antagonist at both peripheral and central muscarinic receptors, is the most valuable of the tropane alkaloids. The mode of action of tropane alkaloids is based on their binding to muscarinic acetylcholine receptors, thus preventing the binding of acetylcholine. Depending on the specificity and selectivity of the muscarinic acetylcholine receptors in different organs, the functions of the smooth muscles and exocrine gland cells as well as the heart rate, respiration and the central nervous system can be modulated [53].

### 2.2. Solanaceae Glycoalkaloids—Physiological Effects in Insects

**Figure 2:**
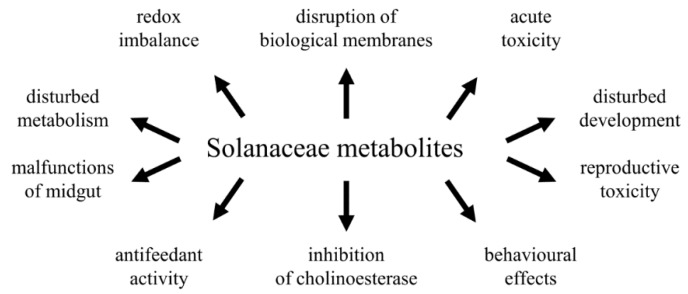
A range of toxic effects of *Solanaceae* metabolites on various levels of biological organization.

*Solanaceae* alkaloids are used by plants as chemical weapons against herbivores, and they have a broad range of biological activity, both towards various species or through various toxicological effects. As shown in Table 1 and Table 2, numerous substances and extracts exhibit insecticidal activity against various insect species and such effects have most often been reported for α-tomatine, α-chaconine, α-solanine and various *Solanum spp*. extracts. It is possible that the most research has been performed on tomatoes and potatoes due to the economic importance and availability of these species. However, acute toxicity has also been reported in plant extracts belonging to other genera, such as *Piper*, *Datura* and *Withania*. This finding proves that plant species remain a rich source of agrochemically important substances. Very often, complex, multidirectional effects of alkaloids are observed, and these may be due to the universal rather than tissue- or cell-specific effects of these compounds. *Solanaceae* alkaloids exert both lethal and sublethal effects and their toxicity is manifested at all levels of biological organization (Figure 2). The low specificity of plant-derived substances is also demonstrated by the wide range of susceptible animals, and toxicity to various orders has been demonstrated within the *Insecta*. Furthermore, α-Chaconine, α-solanine and various *Solanum sp.* extracts have been shown to be toxic to leaf-eating insects, pests of stored products (e.g., seeds, flour), mosquitos that feed on animal tissues, termites or flies and cockroaches that feed on feces and garbage and predatory species (Table 1 and Table 2).

Of course insects develop resistance against toxicants, and herbivores are more prone to developing resistance to plant defenses due to natural selection than predatory or parasitoid insects, which are natural enemies of pests [54,55]. The alkaloids present in the bodies of pests may negatively affect their predators [56,57], and this phenomenon may seriously disturb biological control and influence IPM strategies. Therefore, detailed research on any new pesticidal substance must be carried out to test effects on non-target fauna.

In the agricultural economy, the ability to rapidly and completely eradicate pests from fields is the most important feature of pesticidal substances, but from the ecological point of view, the use of highly toxic pesticides may not be the best solution. A compromise can be achieved using substances that can decrease insect reproduction or that have antifeedant activities. As a consequence, they can significantly limit the pest population within the exposed agroecosystem, and under current IPM strategies, such agents should have priority over acutely toxic chemical insecticides. Secondary plant metabolites are among the major chemical weapons produced by plants against herbivores; they deter them from feeding on leaves or unripe fruits. Therefore, there are many reports demonstrating their antifeedant activity, and such effects have been proven for *L. decemlineata*, *Tribolium castaneum* (Herbst), *Helicoverpa armigera* (Hubner) and some *Spodoptera* species (compare Table 1 and Table 2). However, herbivorous insects develop resistance to the chemicals produced by plants. Hori *et al.* [58] reported no feeding effect of α-solanine, α-chaconine and α-tomatine on an herbivorous lady beetle, *Henosepilachna vigintioctomaculata* Motsch, but the authors observed decreased feeding when these insects were exposed to nicotine and capsaicin, substances obtained from non-host plant species. Similarly, *S. tuberosum* leaf extract has cardiotropic effects on *Zophobas atratus* (Fab.) but not *L. decemlineata*, [59], indicating an evolutionary adaptation of Colorado potato beetles to potato glycoalkaloids. Therefore, just like chemical insecticides, bioinsecticides have to be carefully managed, and their activities against various target and non-target species should be studied before field application.

Apart from feeding, alkaloids affect other crucial physiological processes, such as the functioning of the cardiovascular system. As was proved quite recently [59,60], different glycoalkaloids significantly affect heart contractile activity in insects. Using the *Z. atratus* beetle heart as a model we showed that glycoalkaloids extracted from potato, tomato and black nightshade inhibit heart contractile activity in a dose-dependent manner. Moreover, the effects caused by extracts were more potent than the individual synthetic glycoalkaloids tested. These results suggest that some synergistic effects may occur between the main glycoalkaloids in tested extracts [60], which would allow the purification procedure to be omitted and reduce the cost of potent bioinsecticides. Moreover, the effects were also observed *in vivo* when *Z. atratus* pupae were injected with the tested substances [60]. Recent studies using the *Tenebrio molitor* heart as an experimental model (Marciniak, unpublished) have revealed that most cardioinhibitory effects are not species-specific.

From an agroeconomic point of view, decreased reproduction (lower number of laid eggs and lower fertility and fecundity) is one the most important insecticidal effects, and malfunctions and malformations of the reproductive system can be induced by glycoalkaloids. The disruptive toxic effects of *Solanaceae* plants on insect reproductive systems have been shown; insects reared on non-host plant species revealed diminished production of oocytes and increased reabsorption of formed oocytes, which inhibits the maturation of eggs [61]. These authors postulate that the glycoalkaloids present in the leafs of non-host plant species may interact with the endocrine system of pests, especially altering juvenile hormone-like activity, similarly to other glycoalkaloids [62], thus disturbing insect development. Similarly, tomatine was reported to synergistically increase 20-hydroxyecdysone activity [63], and the chemical structure of aglycone is highly similar to the structure of 20-hydroxyecdysone, which is a hormone that regulates insect molting and metamorphosis. Glycoalkaloids, the insect hormone hydroecdysone and the other chemical compounds are derived from sterols, so they have very similar chemical structures. Hence, glycoalkaloids, through their similarity to sterols, may affect insect molting and development processes that are regulated by steroidal hormones such as ecdysone. Plants often produce substances that mimic insect hormones [64,65,66], and as a consequence, they disturb insect molting and development to limit the losses in plant organs caused by pests. Hence, an increased amount of glycoalkaloids within pest-infested tissues not only causes acute toxicity but also alters molting and disturbs insect metabolism so that less plant material is eaten.

#### Effects of Alkaloids on Insect Cells and Tissues

There are a number of records of *Solanaceae* secondary metabolite activity at the cellular or subcellular level. These compounds mainly disturb the structure of biological membranes and cellular metabolism.

Potatoes and other *Solanaceae* plants contain, *inter alia*, glycoalkaloids, a family of steroidal compounds that can act as cellular membrane-disrupting factors or inhibitors of acetylcholinesterase activity [45,67]. Nicotine obtained from *Nicotiana spp*. exhibits high insecticidal activity, mostly because it mimics acetylcholine and intensifies synaptic transmission. Homogenates from several insect species were assayed for acetylcholinesterase inhibition by the potato glycoalkaloid α-chaconine. Colorado potato beetle acetylcholinesterase was up to 150-fold less sensitive than that of the other species tested, and acetylcholinesterase from an insecticide-resistant strain of Colorado potato beetle was more sensitive to inhibition than the susceptible strain. Most of the tested insect species had inhibitory concentrations that caused a 50% reduction in activity in the range of 5 to 40 μM. The sensitivity of various isoforms of insect acetylcholinesterases was similar to that of mammalian cholinesterases in their response to α-chaconine. The results indicate that pesticides and host plant resistance factors may interact at the same target, so changes in the target due to selection pressure from either pesticides or host plant resistance factors could affect the efficacy of both control strategies [68].

The second cellular metabolic pathway that is affected by a variety of allelochemicals, including alkaloids, is the antioxidant system. Alkaloids, after penetrating an organism, lead to the generation of reactive oxygen species (ROS), which cause oxidative stress that results in processes such as the peroxidation of membrane lipids, the disruption of mitochondrial membrane potential or protein damage. The generation of ROS promotes antioxidant mechanisms such as the activation of the antioxidant enzymes glutathione S-tranferase (GST), catalase or superoxide dismutase [69].

Recently, we reported that dietary α-solanine treatments influence the biological fitness of wax moth, *Galleria mellonella* (L.), larvae [23,69], and the mechanism appears to be related to the effects of α-solanine–induced oxidative stress on the antioxidant systems, as shown by the influence of this glycoalkaloid on malondialdehyde (MDA) and protein carbonyl (PCO) contents, changes in GST enzyme activity, and declines in important life-table parameters. Lipid peroxidation and protein carbonylation levels, as measured by MDA and PCO concentrations, vary across tissues and α-solanine concentrations, respectively. Based on studies of insect species exposed to pro-oxidant allelochemicals [70,71,72,73], we can speculate that the α-solanine-induced MDA and PCO content in the midgut and fat body followed from ROS production and reduced non-enzymatic protection from pro-oxidants during exposure to α-solanine. Decreased GST activities in the fat body and increased GST activities in midgut tissue are consistent with increased rates of adaptive metabolic responses to elevated lipid peroxidation and protein oxidation [23]. However, the midgut seems to be relatively resistant to various xenobiotics, and we previously showed that midgut cells present a lower level of malformations, with a lower increase in antioxidant enzyme activities, than the fat body, which showed much more intensive malformations, even though the activity of the antioxidant enzymes was higher than in the gut [23]. We think that this phenomenon is connected to the relatively short exposure time to xenobiotics in moving food and the rapid transfer across the gut to other organs.

The mechanism of damage may be a quantitative issue in which the amounts of pro-oxidants in the diets appear to overtake the protective mechanisms, leading to long-term oxidative stress. However, Krishnan and Sehnal [72] did not observe significant alterations to antioxidant defense systems in the gut of *Spodoptera littoralis* exposed to 0.1% α-solanine. Additionally, the antioxidant activity of the potato tuber is not directly correlated to the amount of α-solanine and α-chaconine but, rather, to phenolic compounds [40]. On the other hand, extracts prepared from different *Solanaceae* species have also been described as antioxidants [74,75,76]. This finding proves that the exact mechanism of action remains to be revealed.

Apart from influencing cellular metabolism, some alkaloids have been proven to disturb cellular structure, and there are numerous data concerning the cytotoxic activity of plant-derived substances, mostly in mammals, because of the possible application of these compounds in cancer treatments [77,78]. For example, α-tomatine binds to sterols present in biological membranes, so it disturbs their biosynthesis and metabolism [79]. Additionally, α-chaconine disturbs biological membranes, which obviously results in cell damage. Furthermore, Mandimika *et al.* [80] observed concentration-dependent leakage of the lactate dehydrogenase from the cytosol of the intestinal epithelial cell line which indicates a disturbance to cell homeostasis due to cell membrane disruption. The authors suggest that α-chaconine can be cytotoxic at a concentration of 20 μM, and Sucha *et al.* [81] reported a lower mitochondrial membrane potential in tumor cells exposed to α-solanine. This glycoalkaloid opened membrane channels, which increased the concentration of calcium ions in cells and led to apoptosis. In contrast, α-solanine was reported as not being active against *Trypanosoma brucei* [82], and similarly, a single application of α-tomatine does not induce apoptosis in the human breast adenocarcinoma cell line [81]. Its activity strongly depends on the amount of cholesterol in the cell membrane, and therefore, the authors suggest that α-tomatine cytotoxicity results from its binding to membrane cholesterols and altering membrane chemical characteristics. Additionally, the saponins present in *Solanaceae* affect cell ultrastructure, including biological membranes [83], and therefore, this activity enables other compounds to enter the cells and affect metabolism at the subcellular level, both ultrastructurally and enzymatically. Finally, Oberdorster, Clay, Cottam, Wilmot, McLachlan and Milner [63] reported on the cytotoxicity of tomatine, by inhibition of cell growth and division, via a pathway that does not involve ecdysone receptors.

## 3. *Solanaceae* Secondary Metabolites as Bioinsecticides—Future Directions

As shown in Table 1 and Table 2, the secondary plant metabolites cause both lethal and sublethal toxic effects, and although acute lethality provides rapid, successful protection of harvested plants against pests, sublethal effects are also very important. Such effects are likely to cause significant changes in exposed populations but over a longer period of time. For example, a decrease in the number of eggs laid leads to a lower number of pests in the next generation, so predation pressure on the population of herbivore pests increases, which may reduce its size. Malformed insects are often weaker, cannot successfully protect themselves against predators, and are more susceptible to bacterial or viral diseases. Furthermore, weak effects observed in the laboratory may be pronounced under environmental conditions. Hence, secondary plant metabolites may play important roles as pesticides, but they may also lead to a decrease in the use of the chemical insecticides, which are currently used in high concentrations, especially against massive outbreaks of pest species.

It is important to note that not only pure substances cause lethal and sublethal effects; water and/or alcohol extracts have revealed various effects against insects. Extracts contain various substances that may act synergistically, and such a synergy was described for *Helix aspersa* snails, in which peel extracts inhibited feeding more effectively than solutions of pure glycoalkaloids [157]. Synergism is not limited to increasing the intensity of the effects caused by the components of extracts. Tak *et al.* [158] showed that the penetration of components is also better when they are applied as a mixture. Moreover, individual compounds can even be inactive when used separately [159]. For instance, α-Chaconine and α-solanine may both decrease insect feeding [25,101,112,160], delay development [21,23,24], affect reproduction and alter enzyme activity [23,47], and kill the pests [113], but the amount of each of those two substances needed to cause the same effect varies [160]. The same sublethal effects could have been observed when *Solanum spp.* extracts were used [133,145,148,149,152], but authors usually report only lethality to various insect stages as it is considered the most important effect. Therefore, the various substances present in extracts may reciprocally increase their toxic effects.

Toxicity tests of plant extracts are also crucial to the process of searching for new, active substances that may be synthesized and used commercially in the future. The standard procedure that leads to the discovery of new insecticides consists of numerous steps. Routine research, extraction and tests of crude extracts begin the procedure, which is finished by finding and describing new insecticides derived from natural products (for comparison, see Pino *et al.* [161]). Therefore, studies examining the activity of extracts are very important as they suggest which species are potential sources of substances with pesticidal activities and point out the target organisms, and they provide suggestions concerning the mode of action of these substances. Moreover, the history of pesticides is directly connected with the use of plant crude extracts or powders. Nicotine extracted from tobacco (*Nicotiana spp.*) was used to control pests as early as the 17th century; unfortunately, this alkaloid is highly toxic to mammals. Therefore, its use as an insecticide should be discussed in a historical context. Perytreum has been used as an insecticide since the beginning of the 19th century [162], and currently, hundreds of plant species are used as insecticides in India [163]. Observations of the biological activity of extracts gave rise to many of the pesticides in current use. Plant-derived poisons have been used as insecticides for hundreds of years [164], and *Nicotiana* species gave rise to many toxic substances. Moreover, pure substances as well as plant extracts are also used as important and fully acceptable medicines [165]. Hence, studies of the biological activities of crude extracts should be appreciated.

From an economic point of view, the use of extracts is also very important. Klonsky [166] analyzed the costs of organic and conventional farming for nine crops, and although the total cost of plant production was significantly higher in organic farming, the cost of pest control was higher in conventional farming. For example, pest control on alfalfa, tomato and broccoli was estimated as being approximately 37%, 11% and 94% more expensive on conventional farms, respectively. Of the tested plant species, only two tree nuts, almonds and walnuts, were more expensive when farmed organically. Amoabeng *et al.* [167] compared the costs and benefits of some synthetic and botanical insecticides, including tobacco, and the use of both types of insecticides resulted in significantly increased incomes. It is noteworthy that the incomes from tobacco-sprayed fields were similar to those sprayed with synthetic insecticides. However, the amount of secondary metabolites in plant tissues changes and depends in various factors such as the organs from which they are collected, the developmental stage of the plant and the presence of a pest infestation [67]. Nevertheless, using botanical insecticides in rotation with conventional pesticides or in mixes to reduce the application of synthetic insecticides or using them alone allows us to decrease the cost of plant protection significantly [168]. Moreover, botanical extracts also have other advantages. First of all, extraction is relatively easy compare to the production of commercial pesticides. Next, *Solanaceae* plants grow in various parts of the world, both naturally and under cultivation, so plants of interest can usually be obtained from the local environment without requiring much money for purchase or storage. Hence, there is growing interest in the properties of extract activities, especially in developing countries where native plants are found that are rich in secondary metabolites. Despite the fact that the amount of secondary metabolites in plants may vary and the lethality of extracts may be weaker than that of the commercial, synthetic insecticides, natural products may be used more often. Furthermore, one cannot underestimate the importance of sublethal effects. Decreased feeding or limited oviposition due to deterrence lead to increased crop production, and sublethal effects pose less of a threat to non-target insects, thus limiting the environmental impacts in contrast to the acute, lethal toxicity of synthetic insecticides. Moreover, the synergistic activity of both groups of insecticides when used together is highly possible, and such a strategy could be used to decrease the amount of chemicals released into environment. However, one must remember that insects may become or already be resistant to various substances, especially those on which they feed [59,160]. Additionally, non-target species may be susceptible to plant-derived insecticides.

In summary, there is a need for more detailed studies of the insecticidal activities of plant-derived substances and plant extracts against various invertebrate species, including economically important pests, other model organisms, beneficial species and non-target animals that might be exposed accidentally, such as soil fauna or aquatic organisms. Careful application of plant-derived substances may become a very important strategy for crop protection. Furthermore, the *Solanaceae* contain numerous substances that may be sources of commercially synthesized pesticides, so extract research is a crucial step in the search for new pesticides. Among those tested, *Nicotiana*, *Solanum*, *Lycopersicon* and *Capsicum* seem to be the most promising genera, and their lethal and various sublethal effects have been described. They affect insects that are pests of various crops and various products. Therefore, we believe that incorporating plant-derived insecticides into IPM strategies may be beneficial to both the human economy and the environment.

## Figures and Tables

**Table 1:** Reported acute and subacute effects of *Solanaceae* pure compounds on insects.

Substance/Extract	Insect Genus/Species	Feeding *	Activity	EC_50_/LC_50_ **	Ref.
(2S,4R)-4-hydroxy-1-methyl-2-pyrrolidine carboxylic acid	*Liriomyza trifolii* Burg.	C	inhibition of oviposition, deterrence	3.7–16.0 µg/cm^2^	[84]
4-amin-1-β-D-ribofuranosyl-2(1*H*)-pirimidinone					
4-aminobutanoic acid					
7-*O*-β-D-apiofuranosyl-(1→2)-β*-*D-glucopyranoside					
2-undecanone	*Aphis craccivora* Koch	C	increased adult mortality	0.48 μmol/cm^2^	[85]
2-dodecanone				0.32 μmol/cm^2^	
2-tridecanone				0.22 μmol/cm^2^	
2-pentadecanone				0.22 μmol/cm^2^	
anabasine	*Apis mellifera*	O	antifeedance	2-25 ppm	[86]
	*Spodoptera litura* (Fabricius)	C		60 µg/cm^2^	[87]
	*Leptinotarsa decemlineata* Say			50 µg/cm^2^	
atropine	*Spodoptera litura* (Fabricius)			50 µg/cm^2^	
	*Leptinotarsa decemlineata* Say			7.38 µg/cm^2^	
atropine	*Lymantria dispar* L.	T	antifeedance, deterrence	4.39 nM	[88]
nicotine				15.6 nM	
				28.3 nM	
scopolamine	*Apis mellifera*	O	deterrence	0.03%	[89]
hyoscyamine			deterrence	0.005%	
			lethality	0.1%	
calystegine B_4_ (1α,2β,3α,4α-tetrahydroxy-*nor*-tropane)	*Bombyx mori* L.,	T	midgut trehalase inhibition	19 μM	[90]
	*Spodoptera litura*	C		40 μM	
capsaicin	*Coptotermes formosanus* Shiraki	O	reduction of the number of microbes: *Spirotrichonympha leidyi*, *Holomastigotoides hartmanni*, *Pseudotrichonympha grassii*, and spirochetes present in the hindgut of a Formosan subterranean termite	0.15–1 ppm	[91]
	*Tenebrio molitor* L.	S	changes in behavioral thermoregulation	10^−7^–10^−4^ M	[92]
	*Myzus persicae* (Sulz.)	C	increased efficiency of synthetic pesticide (neemix, pyronyl, m-pede)	1–10^5^ mg/L	[93]
	*Leptinotarsa. decemlineata*	C	increased metabolic rate, changes in the thermal preferences (preferring lower temperature)	10^−4^–10^−7^ M	[94]
chlorogenic acid, rutin, tomatine	*Heliothis virescens* F., *Manduca sexta* L., *Pseudoplusia includes* Walkler, *Spodoptera frugiperda* Smith	C	extended duration of molting	-	[95]
chlorogenic acid, rutin, tomatine	*Podisus maculiventris*	P	reduced development, weight and growth	5–20 µmol/g of diet	[96]
laxumin A	*Schizaphis graminum* (Rondani)	C	decreased adult survival	4.3 μM	[97]
laxumin B				6.1 μM	
foliumin				137 μM	
solanine				138 μM	
chaconine				137 μM	
tomatine				7.3 μM	
leptine	*Leptinotarsa decemlineata*	C	reduced feeding	8200 µg/g dry weight of leaf	[98]
leptine I	*Leptinotarsa decemlineata*	C	antifeedance, reduced neuronal responses to chemicals that stimulate feeding	0.01–1 mM	[99]
luciamin	*Schizaphis graminum*	C	antifeedance, decreased adult survival	50–500 μM	[100]
nicotine	aphids, whiteflies, leafhoppers, thrips and other (generally non-species specific)	-	mimicked acetylcholine and interacted with nicotinic acetylcholine receptors	-	[101]
	*Apis mellifera*	O	decreased larval survival	50 ppm	[102]
			deterrence, reduced survival	3–1000 µM	[103]
			increased food intake (at low concentrations), decrease food intake (at high concentrations)	2–25 ppm	[86]
			deterrence	0.03%	[89]
			lethality	0.2%	
	*Cotesia congregata*	P	reduced emergence, number of formed cocoons,	0.025–0.1%	[54]
	*Hyposoter annulipes*	P	reduced emergence, number of formed cocoons, longer larval development, smaller adults	0.025–0.1%	
	*Manduca sexta*	C	no lethal effect, decreased larval mass,	0.1% of fresh diet	[104]
	*Spodoptera exigua*	C	lethality, decreased body mass	0.1% of fresh diet	
phytol (2*E*)-3,7,11,15-tetramethyl-2-hexadecen-1-ol	*Liriomyza trifolii*	C	oviposition deterrence	0.1% of fresh diet	[84]
salpichrolide A	*Musca domestica* L.	O	antifeedance	290 ppm	[105]
salpichrolide C				310 ppm	
salpichrolide G				203 ppm	
salpichrolide A, salpichrolide G	*Tribolium castaneum* (Herbst)	S	delay in development stage (from larva to adult)	-	[106]
serine protease inhibitors	*Manduca sexta*, *Spodoptera littoralis* (Fabricius)	C	inhibited digestive herbivore gut proteases	-	[107]
solamargine	*Macrosiphum euphorbiae* (Thom.)	C	deterrence, decreased reproduction rate	50–500 μM	[108]
solamargine, solasonine	*Manduca sexta*	C	inactive	1–3 μmol/g of diet	[21]
solamargine, solasonine, tomatine	*Tribolium castaneum*	S	inhibited larval growth	1–3 μmol/g of diet	
solasodine	*Macrosiphum. euphorbiae*	C	deterrence, lag (delay) in appearance of new-born nymphs	50–500 μM	[108]
	*Tribolium confusum*	S	Malformations of all insect stages, decreased rate of pupations, inhibited metamorphosis, decreased adult survival	0.1%–3.0%	[109]
solasodine, tomatidine, tomatidenol	*Tribolium castaneum*	S	inactive	1-3 μmol/g of diet	[21]
tomatidine	*Macrosiphum. euphorbiae*	C	deterrence and lethal to adults	51.6 mg/L	[24]
solanidine					
α-tomatine	*Manduca sexta*	C	inhibition of larval growth	50–500 μM	[21]
	*Hyposoter exiguae*	P	prolonged larval development; disruption or prevention of pupal eclosion; morphological and anatomical malformations reduction in weight and longevity of adults	12 μmol to 20 μmol/g of diet	[57]
	*Phthorimaea operculella* Zell.	C	negatively and significantly correlated with development rate (head capsule size) of larvae reared in the fruits	-	[110]
	*Drosophila melanogaster*	O	cytotoxic for cell line	0.001-50 μM	[63]
α-chaconine	*Leptinotarsa decemlineata*	C	no effects on survival, induced agitated and restless behavior	-	[24]
	*Plutella xylostella* (L.)	C	ovicidal, highly toxic to deposited eggs	-	
	*Myzus persicae*	C	deterrence, mortality	-	
	*Ceratitis capitata* (Wied.)	C	decreased larval survival, lower pupal weights, extended pupation period, and increased period of adult emergence	-	
	*Empoasca fabae* Harr.	C	decreased nymph survival	-	
α-chaconine	*Myzus persicae*	C	reduced fecundity and feeding of adults, reduced weight, increased mortality of nymphs	0.1-1.6 mg/mL of diet	[111]
	*Pseudoplusia includes*	C	lowered body weight, total weight gain, and larval survival, but not pupal weight	18.1 μg/mg of insect	[25]
α-solanine				22.5 μg/mg of insect	
	*Myzus persicae*	C	reduced fecundity and feeding of adults, reduced weight, increased mortality of nymphs	0.1–1.6 mg/mL of diet	[111]
α-tomatine	*Heliothis zea* (Boddie)	C	decreased food utilization, inhibition of larvae growth	0.3 μmol/g of diet	[112]
	*Spodoptera exigua*	C	no significant antifeedance	1 μmol/g of diet	
α-chaconine, α-solanine	*Henosepilachna vigintioctomaculata* Motsch.	C	stimulated feeding	-	[58]
	*Macrosiphum. euphorbiae*	C	delayed the appearance and decreased the number of nymphs	-	[24]
α-solanine	*Spodoptera littoralis*	C	no significant effects on midgut antioxidant defence system	0.05%–0.1% in diet	[72]
	*Galleria mallonella* (L.)	O	decreased survival of larvae, pupae and adults; decreased fecundity and fertility; increased malondialdehyde and protein carbonyl content in midgut and fat body of larvae; increased activity of midgut glutathione S-transferases and decreased activity of fat body glutathione S-transferases	0.15–15 μg/g of diet	[23]
			increased mortality of larvae, pupae and adults; disturbance of fecundity and fertility; generation of oxidative stress; decrease in glutathione S-transferases enzymatic activity in fat body	3.1 mg/g of diet	[69]
α-solanine	*Tribolium castaneum*	S	acute toxicity (high mortality)	64.8 μg/cm^2^	[113]
α-chaconine				76.4 μg/cm^2^	
α-tomatine				118.0 μg/cm^2^	
α-solanine	*Zophobas atratus*	O	decreased heart activity in pupae and adults	10^−6^–10^−3^ M	[60]
α-chaconine					
α-tomatine					
solamargine					
solasonine					

* Insects were classified as: C—crop pests, T—tree pests, S—stored product pests, P—parasitoids and predators, O—others (incl. mites, termites); ** If EC_50_/LC_50_ was not available, the concentration range was added.

**Table 2:** Reported acute and subacute effects of *Solanaceae* extracts on insects.

Substance/Extract	Insect Genus/Species	Feeding *	Activity	EC_50_/LC_50_ **	Ref.
*Capsicum annuum* L. leaf extract	*Frankliniella occidentalis* (Pergande)	C, T	larvicidal, interruption of next stage development, decreased efficiency of hatched eggs	-	[114]
	*Liriomyza trifolii*	C	ovipositional deterrence	0.15–147 μg/cm^2^	[115]
	*Spodoptera litura*	C	antifeedance, interfered with the molting process and caused morphological abnormalities	0.5–5 mg/cm^2^	[116]
	*Achaea janata* (L.)	C, T			
	*Sitophilus oryzae* (L.)	S	increased adult mortality, deterrence	1.06 mg/g of diet	[117]
	*Tribolium castaneum*	S		1.24 mg/g of diet	
*C. annuum* fruit extract	*Attagenus unicolor japonicas* Reitter	O	weak antifeedance	1.3–5.2 mg/cm^2^	[118]
*Capsicum frutescens* L. leaf extract	*Plutella xylostella*,	C	antifeedance, deterrence, reduced infestation	Cp 3%	[119]
	*Brevicoryne brassicae* (L.)	C			
*Cestrum diurnum* L. leaf extract	*Anopheles stephensi* Sweet & Rao	O	larvicidal	0.70%	[120]
	*Culex quinquefasciatus* Say	O		0.29%	[121]
*Cestrum nocturnum* L. root and leaf extract	*Aedes aegypti* (L.)	O	larvicidal, inhibition of pupal development and adult emergence	15.4 ppm	[122]
*Cestrum parqui* L’Her. leaf extract	*Spodoptera litoralis* (Fabricius)	C	larval morality caused exuviation difficulties, molting disorders, malformations, oviposition inhibition	2%–32% in diet	[123]
	*Ceratitis capitata*	C	toxicity to neonate larvae when ingested through diet, inhibited or delayed larval development and reduced the percentages of pupae formed and adult emergence, diminished adult reproductive potential	0.9%	[124]
*Datura metel* L. leaf extract	*Coptotermes formosanus*	O	larval mortality	298 ppm	[125]
	*Helicoverpa armigera* (Hubner)	C	larval mortality, growth inhibition of the larvae, antifeedance	5.99 μg/cm^2^	[126]
	*Anopheles subpictus* Gr.	O	larvicidal	2.11 mg/mL	[127]
	*Culex quinquefasciatus*	O		-	[128]
*Datura stramonium* L. leaf extract	*Tribolium castaneum*,	S	antifeedance, contact toxicity	3936 mg/L	[129]
	*Corcyra cephalonica* (Stainton)	S		-	
	*Callosobruchus maculatus* (F.),	S	inhibition of intestinal α-amylase activity	0.125–2.0 mg/mL	[130]
	*Rhyzopertha dominica* (F.)	S			
	*Sitophilus granaries* (L.)	S			
	*T. granarium*	S, O			
*Lycium cestroides* Schltdl. leaf extract	*Myzus persicae*,	C	antifeedance, settling inhibition, contact toxicity	0.1 mg/cm^2^	[131]
	*Rhopalosiphum padi* (L.),	C			
	*Epilachna paenulata* (Germ.),	C			
	*Spodoptera littoralis*	C			
*Lycopersicum esculentum* Mill. leaf extract	*Zophobas atratus*	O	decreased heart activity in pupae and adults	10^−6^–10^−3^ M	[60]
	*Spodoptera litura*	C	interfered with molting process and caused morphological abnormalities	3.60 mg/cm^2^	[116]
	*Achaea janata*	C, T		3.81 mg/cm^2^	
*Nicotiana tabacum* L. leaf extract	*Plutella* *xylostella*	C	antifeedance, deterrence	-	[119]
	*Brevicoryne brassicae*	C			
*Nicotiana plumbaginifolia* Viv. leaf extract	*Anopheles stephensi* Liston	O	larvicidal	15.49 ppm	[132]
*Solanum sisymbriifolium* Lam. fruit extract	Anopheles gambiae Giles	O	larvicidal	0.75 g/mL	[133]
	*Anopheles funestus* Leeson	O		0.75 g/mL	
*Solanum elaeagnifolium* Cav. plant extract	*Blattella* *germanica*	O	repellence, antifeedance	50-150 mg/mL	[26]
*Solanum incanum* L. plant extract	*Amitermes messinae* Full.	O	increased adult mortality	-	[134]
	*Microtermes najdensis* Harris	O		-	
*Solanum jasminoides* plant extract	*Phlebotomus papatasi* Harris	O	repellence, larvicidal	-	[135]
*Solanum melongena* L. fruit extract	*Spodoptera litura*	C	antifeedance, inhibited larval growth, interfered with molting process and caused morphological abnormalities, inhibited intestinal serine protease activity n	0.5–5 mg/cm^2^	[116]
	*Achaea janata*	C, T			
*S. melongena* leaf extract	*Sitophilus oryzae*,	S	increased adult mortality, deterrence	0.05–0.5 mg/cm^2^	[117]
	*Tribolium castaneum*	S			
*Solanum nigrum* L. leaf extract	*Culex quinquefasciatus*	O	increased larvae morality	44.97 ppm	[136]
	*Anopheles culicifacies* Christophers	O	larvicidal	208.5 ppm	[137]
	*Culex quinquefasciatus*	O		337.2 ppm	
	*Aedes aegypti*	O		359.0 ppm	
	*Aedes caspius* (Pallas)	O		51.29 mg/L	[138]
	*Culex pipiens* (L.)	O		125.89 mg/L	
*S. nigrum* leaf and green berry extract	*Culex quinquefasciatus*	O	increased first instar larvae morality and also in second and third instar	2.62 ppm	[139]
	*Anopheles* *stephensi*	O		2.12 ppm	
*S. nigrum* plant extract	*Leptinotarsa decemlineata*	C	acute toxicity	Cp 40%	[140]
*Solanum pseudocapsicum* L. leaf and seed extracts	*Agrotis ipsilon* (Hufnagel)	C	antifeedance, larvicidal, deformations in next instar larvae, in pupae and adults (after larvae treatment)	0.625%–5%	[141]
*S. pseudocapsicum*. seed extracts	*Helicoverpa armigera*	C	antifeedance, malformations	0.625–5 mg/L	[142]
*S. pseudocapsicum* seed extracts	*Spodoptera litura*	C			
*Solanum suratense* Burm. plant extract	*Callosobruchus chinensis* (L.)	S	inhibition of oviposition	1%–10%	[143]
*Solanum suratense* Brum. leaf extract	*Culex quinquefasciatus*	O	larvicidal, disrupted molting and metamorphosis, induced malformation, extended larval duration and inhibited adult emergence	23.53%	[144]
*Solanum torvum* Sw. leaf extract	*Anopheles stephensi*	O	larvicidal	29.65 ppm	[145]
	*Culex quinquefasciatus*	O		20.56 ppm	
*Solanum trilobatum* L. leaf extract	*Aedes aegypti*	O	larvicidal, reduced pupation ratio	125.43 ppm	[146]
	*Anopheles stephensi*	O		127.77 ppm	
	*Culex quinquefasciatus*	O		116.64 ppm	
	*Hippobosca maculate* (L.)	O	increased adult mortality	432.77 ppm	[147]
*Solanum tuberosum* L. vegetable waste extract	*Culex quinquefasciatus*	O	larvicidal	1.30%	[148]
	*Anopheles stephensi*	O		1.18%	
*S. tuberosum* leaf extract	*Zophobas atratus* (Fab.)	O	*in vivo* cardioinhibitory activity in pupae	10^−6^–10^−3^ M	[60]
			*in vitro* cardioinhibitory activity in adults	10^−7^–10^−1^ M	[59]
	*T. molitor*	S	non-effect on heart of adult beetle	10^−7^–10^−1^ M	[59]
	*Leptinotarsa decemlineata*	C	increased mortality of larvae, pupae and adults; disturbance of fecundity and fertility; generation of oxidative stress; decreased GST enzymaticactivity in fat body	1 mg/g of diet	[69]
	*Spodoptera exigua*	O			
*Solanum* *verbasicum* leaf extract	*Culex quinquefasciatus*	O	larvicidal	100–1000 ppm	[149]
*Solanum villosum Mill.* fruit extract	*Aedes* *aegypti* (L.)	O	larvicidal	0.1%–0.5%	[150]
	*Culex quinquefasciatus*	O	larvicidal (inhibited larvae growth and pupation)	321.89 ppm	[151]
	*Anopheles subpictus*	O	larvicidal	24.20 ppm	[152]
*Solanum xanthocarpum* Schrad. fruit extract	*Aedes aegypti* (L.)	O	larvicidal and pupacidal activity	170.91 ppm	[153]
*S. xanthocarpum* shoot extract	*Culex quinquefasciatus*	O	contact toxicity, larvicidal and pupacidal activity	155.29 ppm	[154]
*Withania somnifera* (L.) Dunal leaf extract	*Spodoptera litoralis*	C	toxicity, molt disturbances, formation of larval–pupal, pupal–adult intermediates and adultoids	50–100 µg/insect	[155]
*Withania somnifera* plant extract	*Culex pipiens*	O	Reduced hatching, pupation, larvicidal activity	132.6 ppm	[156]

* Insects were classified as: C—crop pests, T—tree pests, S—stored product pests, P—parasitoids and predators, O—others (incl. mites, termites); ** If EC_50_/LC_50_ was not available, the concentration range was added.
